# Do submillisievert-chest CT protocols impact diagnostic quality in suspected COVID-19 patients?

**DOI:** 10.1177/20584601211073864

**Published:** 2022-01-19

**Authors:** Hans-Martin Thieß, Keno K Bressem, Lisa Adams, Georg Böning, Janis L Vahldiek, Stefan M Niehues

**Affiliations:** 1 Department of Radiology, 9164 Charité Universitätsmedizin Berlin Campus Benjamin Franklin, Berlin, Germany; 2 Department of Radiology, 14903 Charité, Universitätsmedizin Berlin, Berlin, Germany; 3 Klinik für Radiologie, 14903 Charité-Universitätsmedizin Berlin, Berlin, Germany

**Keywords:** Lung, COVID-19, low-dose CT, adults, radiation safety, image quality

## Abstract

**Background:**

During the ongoing global SARS-CoV-2 pandemic, there is a high demand for quick and reliable methods for early identification of infected patients. Due to its widespread availability, chest-CT is commonly used to detect early pulmonary manifestations and for follow-ups.

**Purpose:**

This study aims to analyze image quality and reproducibility of readings of scans using low-dose chest CT protocols in patients suspected of SARS-CoV-2 infection.

**Materials and Methods:**

Two radiologists retrospectively analyzed 100 low-dose chest CT scans of patients suspected of SARS-CoV-2 infection using two protocols on devices from two vendors regarding image quality based on a Likert scale. After 3 weeks, quality ratings were repeated to allow for analysis of intra-reader in addition to the inter-reader agreement. Furthermore, radiation dose and presence as well as distribution of radiological features were noted.

**Results:**

The exams’ effective radiation doses were in median in the submillisievert range (median of 0.53 mSv, IQR: 0.35 mSv). While most scans were rated as being of optimal quality, 38% of scans were scored as suboptimal, yet only one scan was non-diagnostic. Inter-reader and intra-reader reliability showed almost perfect agreement with Cohen’s kappa of 0.82 and 0.87.

**Conclusion:**

Overall, in this study, we present two protocols for submillisievert low-dose chest CT demonstrating appropriate or better image quality with almost perfect inter-reader and intra-reader agreement in patients suspected of SARS-CoV-2 infection.

## Introduction

Since December 2019, the severe acute respiratory syndrome coronavirus 2 (SARS-CoV-2), which originated from Wuhan, China, showed a rapid worldwide spread with more than 145 million infections and 3.1 million deaths as of April 2021.^1^ Patients commonly present with several unspecific clinical symptoms, such as fever, dry cough, dyspnea, fatigue, and limb pain, with an unknown number of asymptomatic patients. The continuous increase in new infections and a lack of effective treatment apart from vaccinations, which are still restricted by insufficient supply, demand for fast and reliable methods for early identification of infected patients. This is aggravated by the current spread of new, possibly more infectious SARS-CoV-2-variants,^2^ such as the British (B.1.1.7) and the South African variants (N501Y.V2). Currently, real-time reverse-transcription polymerase chain reaction (RT-PCR) of throat swabs, deep nasal swabs, or sputum represents the gold standard for detecting SARS-CoV-2.^3^ However, chest CT can play a vital role in the initial identification of infected patients for several reasons: First, due to low examination times and fast availability of results, it can bridge the time until RT-PCR results arrive,^4^ thus enabling the early isolation of patients with CT findings typical of COVID-19 preventing further transmission, especially when combined with antigen rapid tests. However, these rapid tests present a widely varying sensitivity depending on which assay is used and current viral load,^5–7^ the latter underlining utility of antigen rapid tests within the first week after symptom onset.^5^ Second, there have been several reports of false-negative results of initial RT-PCR^8–10^ and studies stating a higher (88%–97% vs. 59%–71%,^11,12^) or similar sensitivity albeit low specificity of chest CT compared to RT-PCR.^3^ Thus, patients with negative RT-PCR and positive CT findings may benefit from additional subsequent PCR testing, especially when providing an epidemical exposure history and/or clinical symptoms coherent with SARS-CoV-2 infection. Third, the wide availability of CT compared to remaining regional shortages of RT-PCR kits accentuates its usefulness in the early diagnosis of COVID-19. Apart from its role in the triage of possibly infected patients, CT can also be of use for follow-up to determine the course of the disease, effectiveness of treatment, and possible complications, such as acute respiratory distress syndrome^13^ or abscess. Furthermore, it can help determine additional causes for rather unspecific clinical symptoms, such as lobar pneumonia, emphysema, cardiac insufficiency, and others. There are several findings in chest-CT of patients with COVID-19 considered to be typical, such as bilateral manifestation with multifocal ground-glass opacities and consolidations (predominantly in a basal and peripheral distribution), vascular thickening and a crazy-paving pattern.^14^ These may correlate with different times in the course of the infection, severity of the course of the disease, and necessity of ICU admission.^15,16^ The major drawback of CT is radiation exposure, which can be reduced by using techniques, such as iterative reconstruction (e.g., SAFIRE/ASIR/AIDR) in combination with specific low-dose protocols.^17^ Due to the high examination count of chest-CT during the coronavirus pandemic, we introduced new, dedicated low-dose protocols for COVID-19 diagnostic, which this study aims to analyze regarding image quality, radiation dose, and reproducibility.

## Material and Methods

### Setting

For this retrospective study, we analyzed 100 non-contrast low-dose CT-examinations between April of 2020 and February of 2021. To avoid bias, we randomly selected scans from 50 patients with and 50 patients without confirmation of SARS-CoV-2 infection via RT-PCR from deep nasal or throat swabs. Patients included presented in the emergency department with symptoms suspicious of SARS-CoV-2 infection. This study was priorly approved by the institutional review board (EA4/140/17).

### CT data acquisition

Image acquisition was conducted using either a Canon Aquilion Prime or GE Lightspeed VCT. Exams included in the study were performed only of the thorax, and no additional scans using different parameters were performed. All studies were non-contrast helical scans in supine position, from the diaphragm upwards and—when possible—in deep inspiration, using examination parameters and reconstruction methods as seen in Table 1. The selected parameters were based on our experience with these scanners utilizing CT protocols routinely used for interstitial lung disease imaging focusing on a middle ground between image noise and the lung as a high-contrast organ. All images were obtained on either an 80-row detector (Aquilion Prime, Canon Medical Systems) or a 64-row detector (GE Lightspeed VCT, GE Healthcare Systems). All parameters used are summed up in Table 1.

**Table 1.** Technical parameters of the COVID-19 imaging protocol.

Settings	Canon Aquilion PRIME	GE Lightspeed VCT
Rotation time (s)	0.27	0.35
Tube voltage (kV)	100	100
Tube current (mA)	10–100	10–100
Noise index	27	30
Pitch factor	1.388	1.375
Collimation (n * mm)	80 * 0.5	64 * 0.625
Iterative reconstruction	AIDR 3D^a^ moderate	ASiR^b^ 50%
Reconstruction	0.5 mm Fc01/Fc85	0.625 mm lung/standard

^a^Adaptive iterative dose reduction 3D.

^b^Adaptive statistical iterative reconstruction.

### Data extraction and subjective image analysis

Individual CT dose index (CTDI_vol_) and dose length product (DLP) of each exam was extracted from the DICOM-header. Effective radiation dose was estimated by multiplying DLP by a conversion factor for chest imaging at 100 kV: 0.0144 mSv × mGy^−1^ × cm^−1^.^18^ Two radiological residents with 5 and 2 years of experience in CT imaging (KKB and HMT) independently rated all exams on a Workstation using MERLIN Diagnostic Workcenter by Phoenix-PACS regarding image quality on a Likert scale from 0 to 3 (0: best quality, no limitations in diagnostic value; 1: good quality, only slight impairments; 2: moderate quality, noticeable decrease in diagnostic value; and 3: worst quality, non-diagnostic scan). The evaluation was based on subjective ratings for the parameters image sharpness (defined as delineation of margins, such as bronchial walls or interlobar fissures in not breath dependent lung sections), image noise in lung parenchyma and soft tissue, respectively, as well as ring and other artifacts, for example, those caused by foreign materials. Three weeks after the initial rating, reevaluation of all included scans was performed, allowing for analysis of intra-reader reliability. To allow for additional assessment of organizational/fibrotic changes of lung parenchyma, including inter-reader reliability, both readers furthermore scored scans for the extent of reticulation, honeycombing, and emphysema to the nearest 5% in the following three zones: The upper zone being defined as at or above the aortic arch, the middle zone as between the aortic arch and the confluence of the pulmonary veins, and the lower zone as at or below the pulmonary veins.^19^ The extent of lung fibrosis was then calculated as the mean of the mean extent of reticulation and honeycombing for each zone. Readers only used axial images for ratings yet were allowed to change window settings. Additionally, based on the study by Wang et al.,^20^ presence and extent of pathological findings of the lung, such as consolidations, ground-glass opacities, distribution, and pleural effusion, were noted**;** presence of crazy-paving patterns was not included in accordance with Wang et al.’s results.

### Statistical analysis

Statistical analysis was performed using the R statistical language Version 4.0.0 and the “tidyverse” library.^21^ Quantitative parameters (e.g., patient characteristics, presence/extent of radiological features) were expressed as median value and interquartile ranges. Cohen’s kappa was used for analysis of inter-reader and intra-reader reliability with κ-values characterized as 0–0.20 = poor, 0.21–0.40 = fair, 0.41–0.6 = moderate, 0.61–0.80 = substantial, and 0.81–1.00 = (almost) perfect agreement.^22^ Differences in quality ratings were analyzed depending on RT-PCR results, presence of CT findings typical of COVID-19, and patients’ sex using Pearson’s chi-squared test. A *p*-value of < 0.05 was considered statistically significant.

## Results

### Patient population and radiation dose

One hundred patients were included in this study, 38 females and 62 males, with a mean age of 71 years (IQR: 22.0). Of those subjects, 17 female and 33 male patients were tested positive for SARS-CoV-2 via RT-PCR on the same day equaling 44.7% and 53.2%, respectively. Data about patients’ BMI was available for 40 patients with a mean BMI of 27.2 kg/m^2^ (IQR: 6.3 kg/m^2^).

Median DLP was 29.6 mGy*cm (IQR: 29.5 mGy*cm) for females and 40.7 mGy*cm (IQR: 19.7 mGy*cm) for males, which results in a median effective dose of 0.43 mSv (IQR: 0.42 mSv) for females, 0.59 mSv (IQR: 0.28 mSv) for males, and 0.53 mSv (IQR: 0.35 mSv) overall using a tissue conversion factor of 0.0144 mSv/(mGy*cm) for chest CT.^18^ Median DLP was 36.3 mGy*cm (IQR: 30.6 mGy*cm) using the Aquilion Prime and 39.6 mGy*cm (IQR 14.8 mGy*cm) using the Lightspeed VCT. This results in a median effective dose of 0.52 mSv and 0.57 mSv.

### Radiological findings

**Figure 1.**
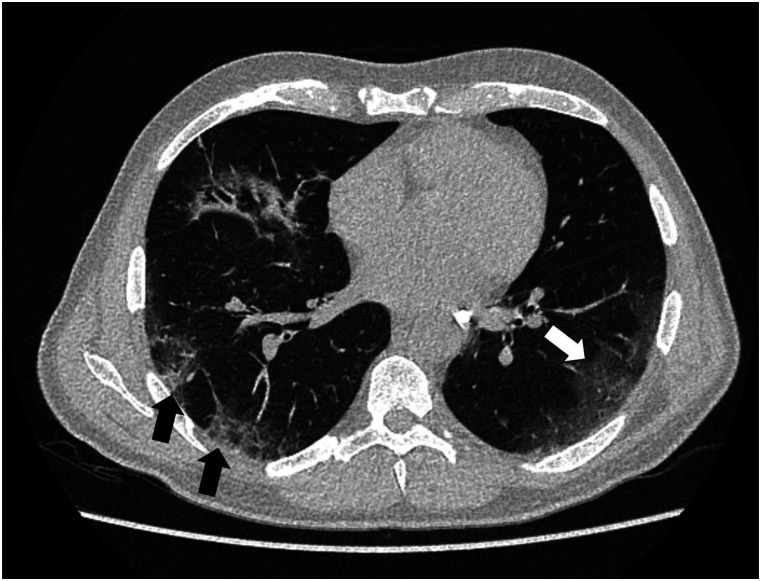
67-year-old male patient with suspected and ultimately confirmed SARS-CoV-2 infection, who presented with fever, dry cough, dyspnea, headaches, and myalgia. Chest CT revealed peripherally accentuated ground-glass opacities (white arrow) and consolidations (black arrows) without pleural effusion. Quality rating: 0 (optimal quality). DLP: 83.4 mGy*cm.

The radiological findings in patients suspected of COVID-19 pneumonia based on positive CT results at presentation are summarized in Table 2. Among these, most scans (51.9%) with ground-glass opacities and/or consolidations showed a peripherally accentuated pattern with basal (31.5%) or dorsal (24.1%) distribution (Figure 1). Pleural effusion was present in only 10 patients (18.5%), and cavitation was seen in one patient. Presence of crazy-paving patterns was not detected in this study, and extent of reticulation was only scored for the purpose of calculating a fibrosis score. The mean extent of fibrosis across the three predefined zones was 2.4%, and the mean extent of emphysema was 0.4%.

**Table 2.** Radiological findings in CT-positive scans.

Findings		Number of scans (54 in total)
Consolidations	Little	26
	Moderate	14
	Severe	2
	Not present	12
Ground-glass opacities	Little	10
	Moderate	30
	Severe	12
	Not present	2
Distribution	Peripheral	29
	Peribronchovascular	8
	Map-like	6
	Focal	6
	Other	5
Accentuation	Basal	18
	Dorsal	13
	Diffuse	11
	Other	12
Atelectasis	In one lobe	3
	In multiple lobes	4
	Dystelectasis	3
	Not present	44
Pleural effusion	Big	1
	Small	9
	Not present	44
Cavitation	Present	1
	Not present	53

### Image quality

**Figure 2.**
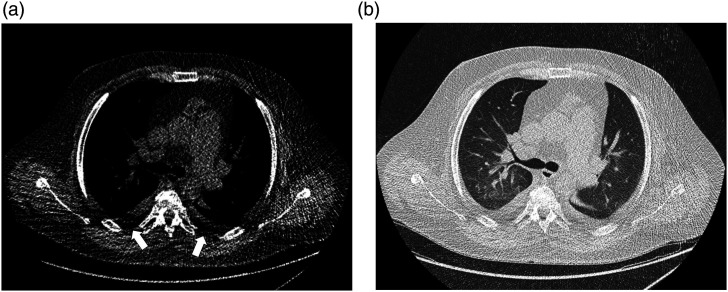
Male patient, 47 years of age, who presented with dyspnea and fever. (a) Chest CT shows minor paravertebral artifacts in surrounding soft tissues (white arrows) resulting in a quality rating of 1 (good quality). (b) Corresponding height in lung window shows no significant artifacts in lung parenchyma. DLP: 40.8 mGy*cm.

**Figure 3.**
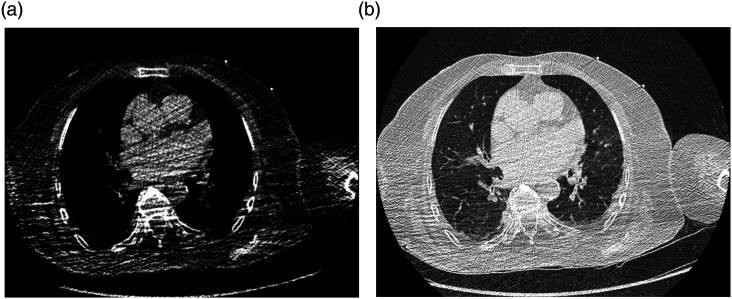
82-year-old female patient, who presented with fever, nausea, and somnolence. (a) Scan with a quality rating of 2 (moderate quality) due to lowered arms with noticeable, dorsally accentuated artifacts. (b) Same scan and position in lung window shows no major artifacts in lung parenchyma. DLP: 34.7 mGy*cm.

**Figure 4.**
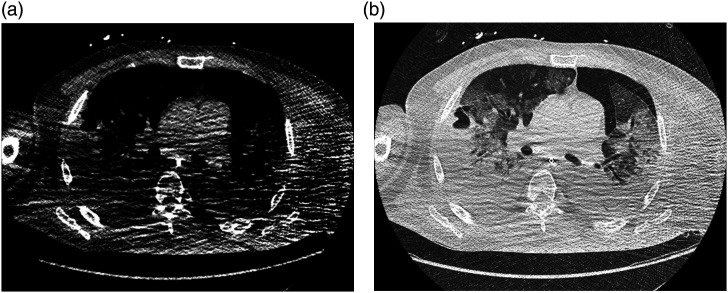
49-year-old male patient, who presented with progressive dyspnea and fever. (a) Chest CT with lowered arms and major artifacts throughout the entire thorax resulting in a quality rating of 3 (non-diagnostic). (b) Lung window at the same position shows minor artifacts in the lung parenchyma. DLP: 45.9 mGy*cm.

The majority of all scans were rated unrestricted positive (Likert score = 0, *n* = 62). 32 patients had slight limitations (Likert value = 1). For those rated impaired (*n* = 6, Likert score = 2 + 3), limitations in patient positioning or motion artifacts accounted for the rating (see Figures 2–4) and only in three cases due to foreign bodies (internal or external). These artifacts primarily impaired image quality of soft tissues while only mildly affecting the evaluation of lung parenchyma (see Figure 3). The second most common reasons for decreased ratings were a lack of sharpness (e.g., blurriness of the interlobar fissure in the upper thorax) or high noise (grainy appearance of mediastinal/subcutaneous adipose tissue or air-filled structures), the latter too predominantly affecting the evaluation of soft tissues. In a few cases, distinguishing pleural effusions from atelectasis was hardly possible, and one scan showed movement artifacts throughout the entire chest, leading to reduced ratings. Only one scan was evaluated as non-diagnostic (score = 3) by both raters due to excessive artifacts throughout the complete scan caused by lowered arms (Figure 4). Table 3 sums up the reasons for reduction of image quality ratings.

**Table 3.** Reasons for reduced image quality ratings (multiple answers possible per scan).

Reason for a reduction in image quality rating	Number of scans affected
Artifacts	24
Caused by lowered arms	6
Caused by foreign bodies	3
High noise	15
Lack of sharpness	7
Pleural effusion indiscernible from atelectasis	2
Movement artifacts	1

There was no significant difference in image quality ratings depending on the result of RT-PCR (*p* = .09). We were neither able to show a significant difference between quality ratings depending on the patient’s sex (*p* = .44) nor findings in chest CT (*p* = .50).

### Inter- and intra-reader reliability

Inter-reader reliability for the assessment of image quality was almost perfect with a κ value of 0.82 (*p* < .001) for all scans and substantial for scans of PCR-positive patients (κ = 0.79, *p* < .001). Analysis of intra-reader reliability demonstrated similar results with a κ value of 0.91 (*p* < .001) for patients with confirmed SARS-CoV-2 infection and 0.82 (*p* < .001) for patients without confirmed disease. Thus, the overall intra-reader reliability of all scans was almost perfect too, with a κ value of 0.87 (*p* < .001). Subgroup analysis, based on which scanner was used, showed substantial agreement (κ = 0.76, *p* < .001) for inter- and almost perfect agreement (κ = 0.91, *p* < .001) for intra-reader reliability for the Canon Aquilion Prime compared to almost perfect agreement (κ = 0.81, *p* < .001) for inter- and substantial agreement (κ = 0.71, *p* < .001) for intra-reader reliability for the GE Lightspeed VCT.

Assessment of the extent of lung fibrosis and emphysema showed almost perfect and perfect inter-reader reliability with a κ of 0.995 and 1, respectively (*p* < .001).

## Discussion

During the COVID-19 pandemic, reliable and readily available diagnostic methods to identify infected patients are in high demand. Chest-CT, being one of those methods due to its frequent use in patients with suspected COVID-19 pneumonia, makes it imperative to reduce radiation dose during scans as much as reasonably achievable, in accordance with the ALARA principle.

In this study, we analyzed two low-dose chest-CT protocols using scanners from two different manufacturers, focusing on possible reductions in image quality and inter-/intra-reader reliability of those ratings.

Using our low-dose protocols, we achieved submillisievert effective doses in both female and male patients. Steuwe et al. reported in their study of 105 patients from a similar, middle-European demographic a mean DLP of 89.3 ± 27.7 mGy*cm. They calculated an effective dose of 1.3 ± 0.4 mSv,^23^ which is substantially higher than our findings due to an increased reference mAs of 60 at 100 kV. Dangis et al. achieved similarly low radiation doses in their study, including 192 middle-European patients, with a mean DLP of 39.9 ± 17.8 mGy*cm resulting in a mean effective dose of 0.56 ± 0.25 mSv.^24^ An even lower radiation dose was reported by Agostini et al.,^25^ who compared nonlow-dose to low-dose chest CT. They stated a median DLP of 19.5 mGy*cm (IQR: 17.5–29.02) and a median effective dose of 0.28 mSv (IQR: 0.25–0.42) for low-dose CT as opposed to 226.2 mGy*cm (IQR: 176.0–322.0) and 3.28 mSv (IQR: 2.55–4.67) for nonlow-dose scans. This resulted in a median dose reduction of 90.6% using long-pitch, dual-source acquisition at 100 kV and an increased reference mAs of 180 with spectral shaping. Spectral shaping using a dedicated tin filter reduces radiation dose by removing a portion of the low-energy photons from the X-ray beam, which usually would not reach the detector and thus does not contribute to image acquisition. This effect was demonstrated by Haubenreisser et al. for chest-CT and by Tan et al., who compared conventional CT-urogram to CT-urogram using a tin filter. Both studies reported a reduction of the effective dose by up to 90%.^26, 27^

Despite the very low radiation doses, our radiologists rated most scans as optimal or slightly suboptimal in image quality, and only a few scans as suffering from limitations in diagnostic value, primarily due to artifacts caused by arms or foreign material within the scan range. Only one scan was rated as being non-diagnostic due to significant artifacts caused by lowered arms. Therefore, in patients, who cannot raise their arms, especially if findings within the soft tissue are of interest to the radiologist, regular chest CT should be considered. The fact that only 62% of scans were given optimal ratings and only a few scans were rated as below average indicates an adequate reduction in radiation dose without loss of diagnostic value. The presented examination technique shows results comparable to Agostini et al. but omits the requirement of spectral shaping and can thus be performed with hardware of other vendors.^25^ However, Agostini et al. did not find significant reductions in diagnostic reliability and evaluation of pathological findings using their low-dose protocol.

Moreover, Agostini et al. also did not report poor or non-diagnostic image quality scans. In their analysis of signal-to-noise ratio (SNR) and contrast-to-noise ratio (CNR), values were overall higher in HD-DECT; however, no significant difference was found when comparing these values for the lung parenchyma. Steuwe et al. presented similar results, stating 13% of low-dose scans as having above average image noise ratings and none as having unacceptable image noise, based on subjective ratings on a five-point Likert scale.^23^ Neither Steuwe et al. nor Agostini et al. analyzed their results concerning inter-reader or intra-reader reliability, the latter due to the low number (10) of included patients.

Inter-reader reliability showed almost perfect agreement in assessing image quality. Three weeks after the first rating, reevaluation too showed almost perfect agreement for patients with and without confirmed SARS-CoV-2 infection, demonstrating the reproducibility of scan results and further underlining reliability of chest-CT in a clinical setting. Inter-reader reliability for assessment of the extent of lung fibrosis and emphysema showed almost perfect and perfect agreement, respectively. Validity, however, is limited due to the patient population not having been selected based on the presence of such chronic changes and thus only few scans demonstrating fibrosis/emphysema. Furthermore, COVID-19-related changes of lung parenchyma, such as ground-glass opacities and consolidations, may mask fibrosis and emphysema.

Radiological findings in our patients are mostly in line with those considered typical of COVID-19, as described by several studies, such as the study by Wang et al. They documented ground-glass opacities, consolidations, or both in all patients undergoing chest-CT, usually in a peripheral (43.6% of subjects) or combined peripheral and central distribution pattern (56.4%).^20,28^ Similar results were reported by Pan et al., who also defined four stages of lung involvement from the early stage (days zero to four) to an absorption stage (≥ 14 days after symptom onset) with an increasing extent of lung opacities until 9–13 days after onset of symptoms and steady decrease thereafter over a time span of in some cases more than 26 days total.^15^

In future research, low-dose scans will be using more recent, AI-assisted reconstruction methods (i.e., AiCE/DLIR). These could additionally be checked for the extent of dose reduction and impact on image quality.

In conclusion, in this study, we demonstrated two protocols for chest-CT with a median ED of 0.53 mSv suitable for examining patients with suspected SARS-CoV-2 infection, which provide reliable scans without significant sacrifices in perceived image quality or diagnostic value.

## Limitations

The major limitations of our study are the retrospective design, including patients from two centers and the limited sample size. Although the data was acquired on devices from two different manufacturers, these results cannot be guaranteed to be transferred to other manufacturers or other devices with different operating or reconstruction software or design. As reviewers were not blinded to PCR results, this may have caused a bias in image quality ratings.

Due to the retrospective design of the study and missing documentation of objective criteria (i.e., scoring) for the clinical severity of COVID-19 in infected patients, we were unable to analyze correlation between clinical severity and image quality ratings or inter-/intra-rater reliability.
